# Mitigation of Synaptic and Memory Impairments through F‐Actin Stabilization in an Alzheimer’s Disease Mouse Model

**DOI:** 10.1002/alz.088101

**Published:** 2025-01-03

**Authors:** P A Haseena, Reddy Peera Kommaddi

**Affiliations:** ^1^ Manipal Academy of Higher Education, Manipal India; ^2^ Centre for Brain Research, Indian Institute of Science, Bangalore India; ^3^ Centre for Brain Research, Indian Institute of Science, Bangalore, Karnataka India

## Abstract

**Background:**

F‐actin plays crucial roles in establishment and maintenance of synapses including post synaptic density organization, facilitation of vesicle trafficking, anchoring of postsynaptic receptors, and involvement in translational machinery. Proteomic analysis of actin‐interacting proteins revealed the interaction of PSD‐95 with actin in synaptosomes from brain cortex of APP/PS1 mice. PSD‐95 functions as a critical scaffold for the assembly of neurotransmitter receptors at the synapse, playing a pivotal role in regulating synaptic strength and plasticity. PSD‐95 directly interacts with NMDA receptors, whereas its interaction with AMPA receptors occurs through an auxiliary subunit, TARPs. The disruption in PSD‐95 association with actin observed in nine‐month‐old APP/PS1 mice may contribute to the synaptic neurotransmission deficit evident in the AD mouse model. Our objective here is to stabilize the F‐actin using Jasplakinolide to examine the disrupted interaction and assess its impact on synaptic function and memory impairment.

**Methods:**

Nine‐month‐old wildtype and APP/PS1 mice were injected intrathecally with vehicle or Jasplakinolide (0.5µg/mice) and contextual fear conditioning memory was assessed 24 hours later. Following behavioral analysis, synaptosomes were isolated and examined for synaptic F‐actin levels and PSD‐95‐ actin association. Additionally, primary cortical cultures were treated with Jasplakinolide and analyzed for F‐actin intensity, dendritic spine density and AMPA receptor subunit surface localization.

**Results:**

Memory recall and PSD‐95 association with actin is dysregulated in nine‐month‐old APP/PS1 mice compared to wildtype mice. However, injection of Jasplakinolide in APP/PS1 mice rescued the memory recall deficit. Synaptic F‐actin levels and the association of PSD‐95 with actin were restored in APP/PS1 mice received the Jasplakinolide. Primary cortical neurons, DIV21, derived from APP/PS1 mice showed a significant reduction in surface localization of AMPA receptor, F‐actin levels and dendritic spine density while these molecular events are restored by stabilization of F‐actin by Jasplakinolide.

**Conclusion:**

The compelling evidence supporting the stabilization of F‐actin suggests a promising strategy for alleviating synaptic impairments in AD, as it not only restores dendritic spines but also increases the surface localization of glutamate receptors. Our findings on complex interplay between cytoskeleton dynamics and synaptic functions may provide potential therapeutic targets aimed at preserving synaptic integrity and ameliorating cognitive decline in AD.

